# Beyond the liver: well-being as the bridge between fatigue and quality of life in chronic hepatitis B patients

**DOI:** 10.3389/fpubh.2025.1535916

**Published:** 2025-06-13

**Authors:** Caixia Zheng, Chunxiu Zhong, Qingyun Tang, Hongyan Xie, Xinrui Gao, Bing Li, Junhua Yin, Li Wei

**Affiliations:** ^1^Department of Infectious Diseases, The First Affiliated Hospital of Xiamen University, Xiamen, China; ^2^Department of Infectious Diseases, Nanfang Hospital, Southern Medical University, Guangzhou, China; ^3^Department of General Practice, Nanfang Hospital, Southern Medical University, Guangzhou, China; ^4^Department of Infectious Diseases, The No.2 People's Hospital of Lanzhou, Lanzhou, China

**Keywords:** chronic hepatitis B, well-being, fatigue, health-related quality of life, mental health

## Abstract

**Background:**

Chronic hepatitis B (CHB) remains a significant public health challenge. Many CHB patients experience fatigue and impaired mental health, affecting their health-related quality of life (HRQoL). This study investigates the relationships between fatigue, well-being, and HRQoL, while identifying risk factors for poor well-being in CHB patients.

**Methods:**

A cross-sectional study was conducted with 428 CHB patients. HRQoL, fatigue, and well-being were evaluated using the 36-Item Short Form Health Survey (SF-36), fatigue was measured using the Multidimensional Fatigue Inventory (MFI-20), and well-being was evaluated using the WHO-Five Well-Being Index (WHO-5). Logistic regression was conducted to evaluate the independent risk factor, and structural equation modeling was performed to explore the relationship between fatigue, well-being and HRQoL.

**Results:**

A total of 315 CHB patients were classified as having high well-being, and 113 as having low well-being, based on WHO-5 scores. Patients in the low well-being group were more likely to be female (26.5% vs. 9.5%, *p* < 0.001), had lower body weight (60.88 ± 9.19 kg vs. 64.47 ± 10.96 kg, *p* = 0.002), and a higher prevalence of cirrhosis (35.4% vs. 21.6%, *p* = 0.004). Well-being scores were positively correlated with all eight HRQoL dimensions, and patients with higher well-being had significantly better HRQoL scores. Conversely, well-being was negatively correlated with fatigue levels, with higher fatigue scores linked to lower well-being. Structural equation modeling showed that fatigue significantly reduced well-being, which in turn decreased HRQoL. Multivariable analysis indicated that independent factors of poor well-being included female gender (OR = 2.692, *p* = 0.004), lower weight (OR = 0.971, *p* = 0.038), lower education level (OR = 0.642, *p* = 0.028), lack of physical exercise (OR = 0.541, *p* < 0.001), and cirrhosis (OR = 1.944, *p* = 0.017). For patients with cirrhosis, only female gender (OR = 5.884, *p* = 0.007) and lack of exercise (OR = 0.541, *p* = 0.023) were significant factors.

**Conclusion:**

Well-being mediates the relationship between fatigue and HRQoL in CHB patients, underscoring its critical role in improving patient outcomes. Interventions targeting well-being, such as promoting physical activity, may enhance HRQoL and overall mental health in CHB patients.

## 1 Introduction

Chronic hepatitis B (CHB) caused by HBV remains a significant public health challenge (1). Although the introduction of potent antiviral drugs with high resistance barriers has greatly improved the prognosis of CHB patients, many CHB patients still experience fatigue and impaired mental health (2, 3). Notably, recent international guidelines emphasize that, beyond improving prognosis and reducing the progression to end-stage liver disease, CHB treatment should also aim to enhance patients’ health related quality of life (HRQoL) (4, 5).

Current data on the well-being of CHB patients are limited. The rise of positive psychology and its integration into public health policies has sparked interest in exploring the relationship between patients’ well-being and health outcomes (6–8). Evidence indicates that well-being is positively associated with longevity (8–10). Additionally, studies have shown that well-being is associated with reduced risk of chronic diseases, including cancer and cardiovascular conditions (11–14). This may be particularly relevant for CHB patients given the increased risk of hepatocellular carcinoma (HCC) associated with persistent HBV infection. While the World Health Organization has developed scales to assess well-being level, there is a lack of in-depth research on well-being in CHB patients (15).

Another important yet understudied issue in CHB is fatigue. Fatigue, a subjective sensation of discomfort, significantly affects patients’ ability to perform daily activities and work under normal conditions (16–18). Studies suggest that a certain proportion of CHB patients suffer from fatigue (19, 20). Despite its prevalence and serious impact on daily life, fatigue is often underrecognized and undertreated, leading to worsening symptoms over time. The causes of fatigue in CHB patients are not fully understood but may involve physiological and psychological factors, such as the chronic nature of HBV infection, liver cirrhosis, and accompanying sarcopenia. While some scales exist to assess fatigue in patients with chronic illnesses, the physiological and psychological mechanisms underlying fatigue in CHB remain unclear and require further investigation.

HRQoL is another crucial aspect that has gained significant attention. Improving the HRQoL for CHB patients has become a key treatment goal (21, 22). Achieving this goal requires addressing both physiological and psychological factors. Despite extensive research on the quality of life in CHB, there is a gap in studies exploring the interplay between fatigue, well-being, and quality of life.

Hence, this study aims to investigate the relationships between fatigue, well-being, and HRQoL in CHB patients and construct a theoretical model to evaluate their interactions. Additionally, this study seeks to identify risk factors for poor well-being in CHB patients, providing a foundation for early detection and targeted interventions to improve their mental health and overall quality of life.

## 2 Methods

### 2.1 Study population

This cross-sectional study included participants who were regularly followed up at the Department of Infectious Diseases, Nanfang Hospital, between January 2018 and December 2022. Inclusion criteria were: (1) a confirmed diagnosis of chronic HBV infection, (2) no other severe illnesses, including mental health disorders, (3) age between 18 and 80 years, and (4) the ability to fully comprehend the questionnaire content. The study adhered to the ethical standards of the Declaration of Helsinki, principles of good clinical practice, and the guidelines of the institutional ethics committee. It was approved by the Ethics Committee of Nanfang Hospital (NFEC-2010-93). All participants received detailed study information, and written informed consent was obtained prior to enrollment.

The education levels of patients were categorized as either ≤ 6 years (Primary), 6 to 12 years (Secondary) and more than 12 years (Tertiary). The frequency of regular exercise was classified as none, 1 time/week, or more than 1 time/week, with the stipulation that a session must last at least 30 min to be considered a single instance of exercise.

### 2.2 Transient elastography assessment

Liver stiffness (LS) were measured using transient elastography, performed by a professionally trained technician according to the manufacturer’s guidelines. Ultrasonic attenuation was evaluated at 3.5 MHz using signals provided by the elastography device. Liver stiffness values were reported in kilopascals (kPa). In this study, LS levels was treated as a continuous variable and evaluated for its potential association with well-being.

### 2.3 Data collection

Sociodemographic and clinical data were obtained through patient interviews and review of electronic medical records. Collected variables included age, gender, height, weight, marital status (married, unmarried, divorced), education level (primary, secondary, tertiary), and lifestyle factors such as alcohol consumption (yes/no), cigarette smoking (yes/no), and physical activity frequency (none, once per week, more than once per week). Clinical data, including cirrhosis status and alanine aminotransferase (ALT) levels, were extracted from medical records. All data were anonymized and verified for accuracy prior to analysis. These varibles were included in the bivariable and multivariable regression models to identify potential risk factors for poor well-being.

### 2.4 Questionnaire assessments

In this study, the SF-36 questionnaire (23, 24), the Multidimensional Fatigue Inventory (MFI-20) (25, 26) and the WHO-5 scales (15, 27) were adopted to evaluate HRQoL, fatigue levels and well-being levels among CHB patients.

The SF-36 questionnaire was used to assess HRQoL across eight domains: physical functioning, role-physical, bodily pain, general health, vitality, social functioning, role-emotional, and mental health. Each domain is scored from 0 to 100, with higher scores indicating better HRQoL in the respective area. Additionally, the SF-36 questionnaire introduces a new dimension: patients’ self-assessment of changes in their quality of life. This dimension is categorized into five levels: much better, somewhat better, same, somewhat worse, and much worse. In this sample, the Cronbach’s alpha coefficient for the SF-36 was 0.79. Furthermore, the physical component summary (PCS) and mental component summary (MCS) scores were calculated according to the standard scoring algorithm provided in the SF-36 manual. Each of the eight subscales was first standardized using *z*-scores based on population norms, then weighted by factor coefficients, and aggregated to generate the PCS and MCS scores. Higher scores indicate better physical and mental health status, respectively.

The MFI-20 is a validated self-report tool for assessing fatigue across five dimensions: general fatigue, physical fatigue, mental fatigue, reduced motivation, and reduced activity. Each dimension is evaluated using specific statements, allowing for a comprehensive assessment of physical and psychological fatigue. Higher scores on the MFI-20 indicate greater levels of fatigue. In this study, the Cronbach’s alpha of the MFI-20 was 0.73.

The WHO-5 Well-Being Index, a brief self-reported questionnaire, was employed to evaluate subjective well-being. It consists of five simple statements addressing positive mood, vitality, and general interest, with higher scores reflecting better well-being. The WHO-5 is widely recognized for its reliability in clinical and research applications. The raw score is calculated by summing the five responses, yielding a range from 0 to 25. Multiplying the raw score by 4 produces a final well-being score between 0 and 100, with higher scores indicating higher levels of well-being. A raw score of 13 serves as the threshold for categorizing well-being levels, distinguishing between high and low well-being (15). Cronbach’s alpha of WHO-5 was 0.90 in this study.

### 2.5 Statistical analysis

The sample size in this study was calculated based on a two-sided independent samples t-test, with a significance level (*α*) of 0.05 and a statistical power of 90% (*β* = 0.10). According to preliminary survey data, the expected mean difference between groups (Δ) was 10 points, with a standard deviation (*σ*) of 15 points. Based on these parameters, a minimum of 96 participants was required for adequate power. The sample size formula used was:


$$n=\frac{2\sigma^{2}{\left(Z_{1-\alpha /2}+Z_{1-\beta }\right)}^{2}}{\Delta^{2}}$$

Statistical analyses were performed using SPSS (version 20.0, Chicago, IL, USA) and GraphPad Prism (version 8.0.2). Continuous variables were expressed as mean ± standard deviation (SD) and compared using Student’s *t*-test. Categorical variables were presented as counts (percentage) and compared using the chi-square test. Binary logistic regression analysis, including both bivariable and multivariable models, was conducted to explore associations between independent variables and well-being status. Model fitness was assessed using the Hosmer–Lemeshow goodness-of-fit test, which confirmed that the logistic regression models adequately fit the data. A *p*-value of < 0.05 was considered statistically significant. Structural equation modeling, conducted using the AMOS 25.0 (IBM), aimed to unveil the potential mechanism behind the fatigue, well-being and HRQoL. Model fit was evaluated using several indices, including CMIN (chi-squared test), CMIN/DF, GFI (Goodness of Fit Index), NFI (Normed Fit Index), IFI (Incremental Fit Index), TLI (Tucker-Lewis Index), CFI (Comparative Fit Index), and RMSEA (Root Mean Squared Error of Approximation).

## 3 Results

### 3.1 Clinical characteristics associated with high well-being scores

A total of 428 CHB patients were included in this study. Based on the WHO-5 questionnaire criteria, 315 patients were categorized into the high well-being group, while 113 were categorized into the low well-being group. Compared to the high well-being group, the low- well-being group had a significantly higher proportion of women (26.5% vs. 9.5%, *p* < 0.001), lower body weight (60.88 ± 9.19 kg vs. 64.47 ± 10.96 kg, *p* = 0.002), and a higher prevalence of cirrhosis (35.4% vs. 21.6%, *p* = 0.004), as detailed in Table 1.

**Table 1:** Demographics of CHB patients enrolled.

Characteristic	High well-being scores (*n* = 315)	Low well-being scores (*n* = 113)	*p* valve
Age (years)	46.87 ± 9.78	48.02 ± 9.95	0.288
Gender			<0.001
Male	285 (90.5)	83 (73.5)	
Female	30 (9.5)	30 (26.5)	
Height (cm)	168.50 ± 6.13	165.76 ± 6.74	<0.001
Weight (kg)	64.47 ± 10.96	60.88 ± 9.19	0.002
Marriage			0.099
Married	62 (19.7)	22 (19.5)	
Unmarried	248 (78.7)	85 (75.2)	
Divorced	5 (1.6)	6 (5.3)	
Education			<0.001
Primary	17 (5.4)	19 (16.8)	
Secondary	145 (46.0)	56 (49.6)	
Tertiary	153 (48.6)	38 (33.6)	
Physical exercise			<0.001
None	46 (14.6)	40 (35.7)	
1 time/week, *n* %	113 (35.9)	35 (31.3)	
>1 time/week, *n* %	156 (49.5)	37 (33.0)	
Alcohol intake, *n* %			0.321
Yes	109 (34.6)	45 (39.8)	
No	206 (65.4)	68 (60.2)	
Cigarette smoking, *n* %			0.950
Yes	91 (28.9)	33 (29.2)	
No	224 (71.1)	80 (70.8)	
Cirrhosis			0.004
Yes	68 (21.6)	40 (35.4)	
No	247 (78.4)	73 (64.6)	
ALT levels	32.25 ± 20.35	32.00 ± 19.24	0.911
LS levels	7.37 ± 4.34	8.23 ± 5.33	0.126

ALT, Alanine Aminotransferase; LS, Liver Stiffness.

### 3.2 Association between HRQoL and well-being scores

**Figure 1:**
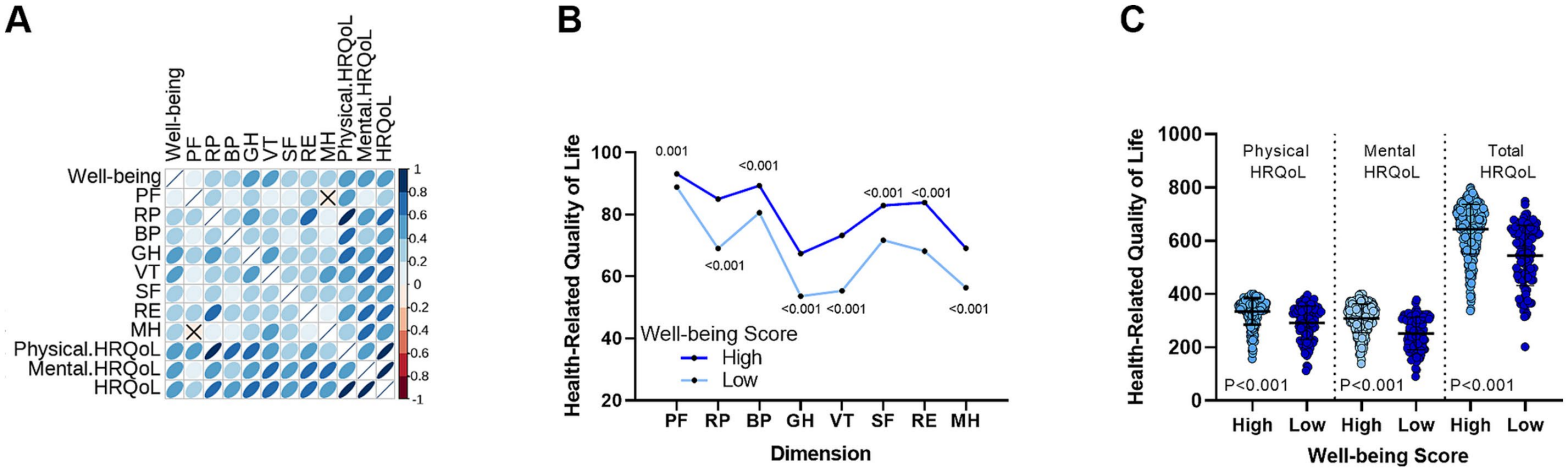
Correlation between well-being levels and HRQoL in CHB patients. **(A)** A significant positive correlation is observed between well-being levels and the eight dimensions of HRQoL. **(B)** Differences in HRQoL scores across eight dimensions between the high well-being group and the low well-being group. **(C)** Comparison of physical, mental, and total HRQoL scores between high and low well-being groups. PF, Physical Functioning; RP, Role-Physical; BP, Bodily Pain; GH, General Health; VT, Vitality; RE, Role-Emotional, SF, Social Functioning; MH, Mental Health.

We analyzed the well-being and HRQoL scores of all CHB patients. As shown in Figure 1A, the well-being scores were positively correlated with the eight dimensions of HRQoL. Furthermore, as demonstrated in Figure 1B, when comparing HRQoL scores between patients with high and low well-being, those with higher well-being showed significantly better scores across all eight dimensions (*p* < 0.001, except PF with *p* = 0.001). Additionally, we examined the differences in physical HRQoL, mental HRQoL, and total HRQoL scores (Figure 1C). Patients with higher well-being had significantly better scores in all three categories compared to those with lower well-being.

### 3.3 Association between fatigue levels and well-being scores

**Figure 2:**
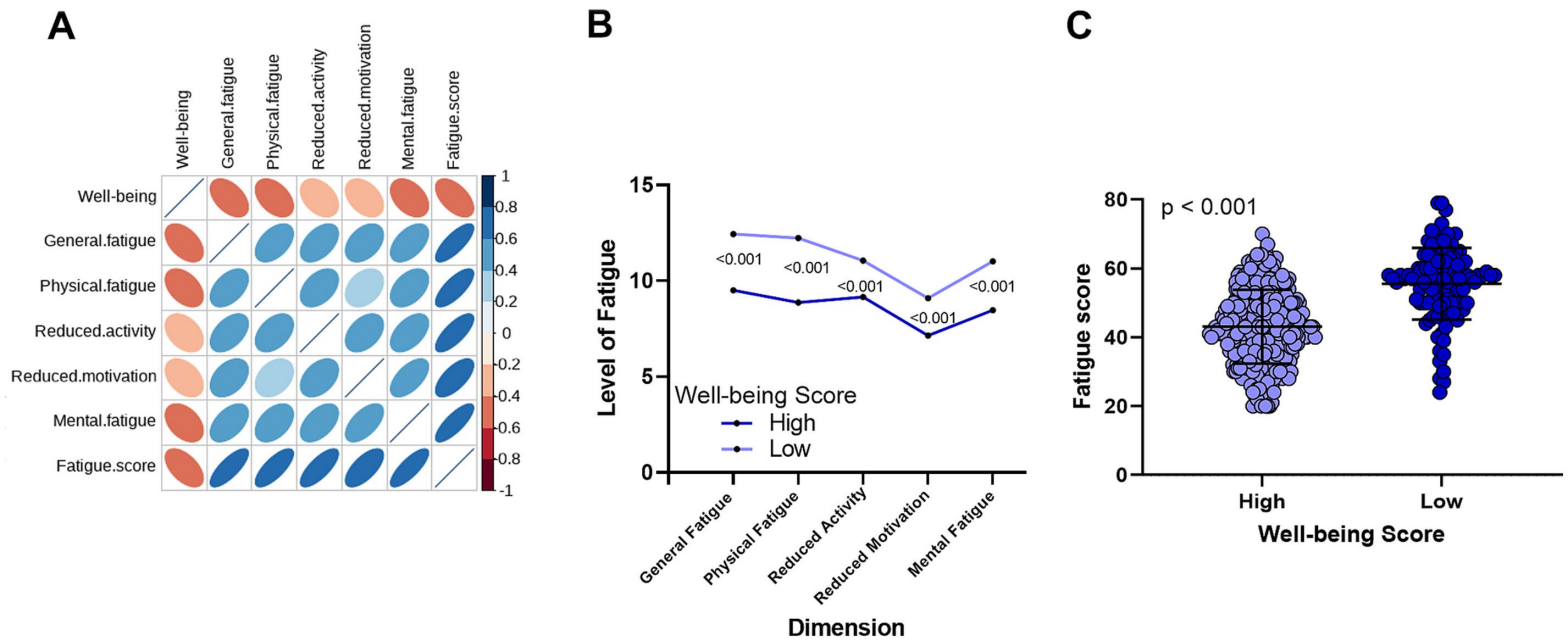
Correlation between well-being and fatigue in CHB patients. **(A)** A significant negative correlation exists between well-being levels and the five dimensions of fatigue. **(B)** Comparison of fatigue levels across five dimensions between the high well-being group and the low well-being group. **(C)** Differences in total fatigue scores between the high well-being and low well-being groups.

Next, we explored the relationship between fatigue and well-being. As shown in Figure 2A, well-being scores were negatively correlated with all five dimensions of fatigue levels —the greater the fatigue, the lower the well-being. Similarly, as illustrated in Figure 2B, patients with higher well-being had significantly lower fatigue scores in all five dimensions compared to those with lower well-being (all *p* < 0.001). Figure 2C further shows that patients with low well-being had significantly higher total fatigue scores.

### 3.4 Association between self-reported health changes and well-being scores

**Figure 3:**
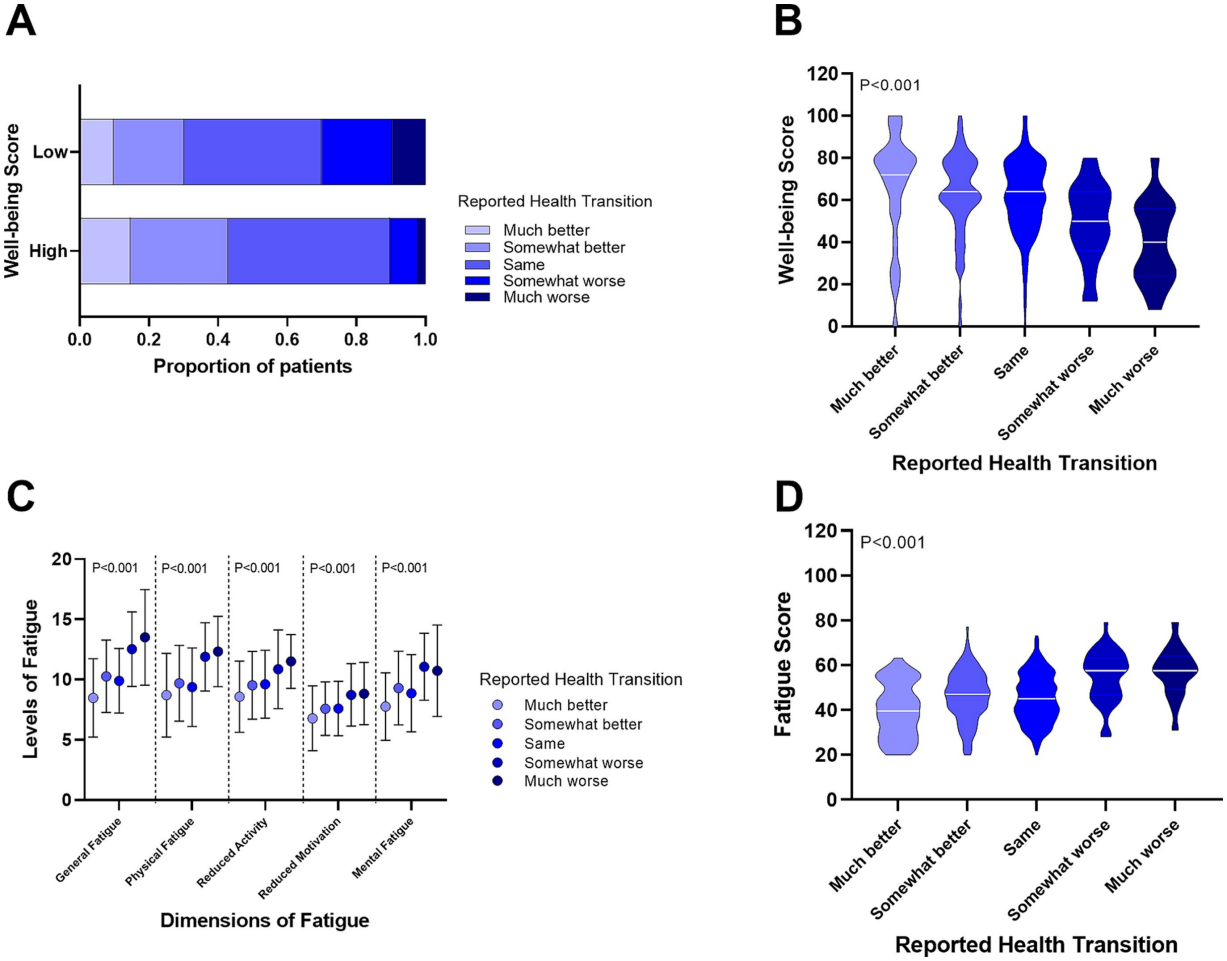
Relationship between self-reported health changes, well-being, and fatigue levels in CHB patients. **(A)** Proportion of self-reported health changes in patients with high and low well-being. **(B)** Differences in well-being scores among CHB patients with varying self-reported health changes. **(C)** Differences in fatigue levels across five dimensions in CHB patients with self-reported health changes. **(D)** Comparison of total fatigue scores in CHB patients with self-reported health changes.

We then assessed the relationship between self-reported health changes and well-being. As shown in Figure 3A, patients with low well-being were more likely to report worse health changes. Figure 3B demonstrates a significant trend, where well-being scores declined progressively from patients reporting “much better” health changes to those reporting “much worse” (*p* < 0.001). Furthermore, we observed that fatigue levels (both in five dimensions and total scores) increased significantly across the same self-reported health change spectrum (Figures 3C,D, *p* < 0.001).

### 3.5 Structural equation model to evaluate the relationship between well-being, fatigue and HRQoL

**Figure 4:**
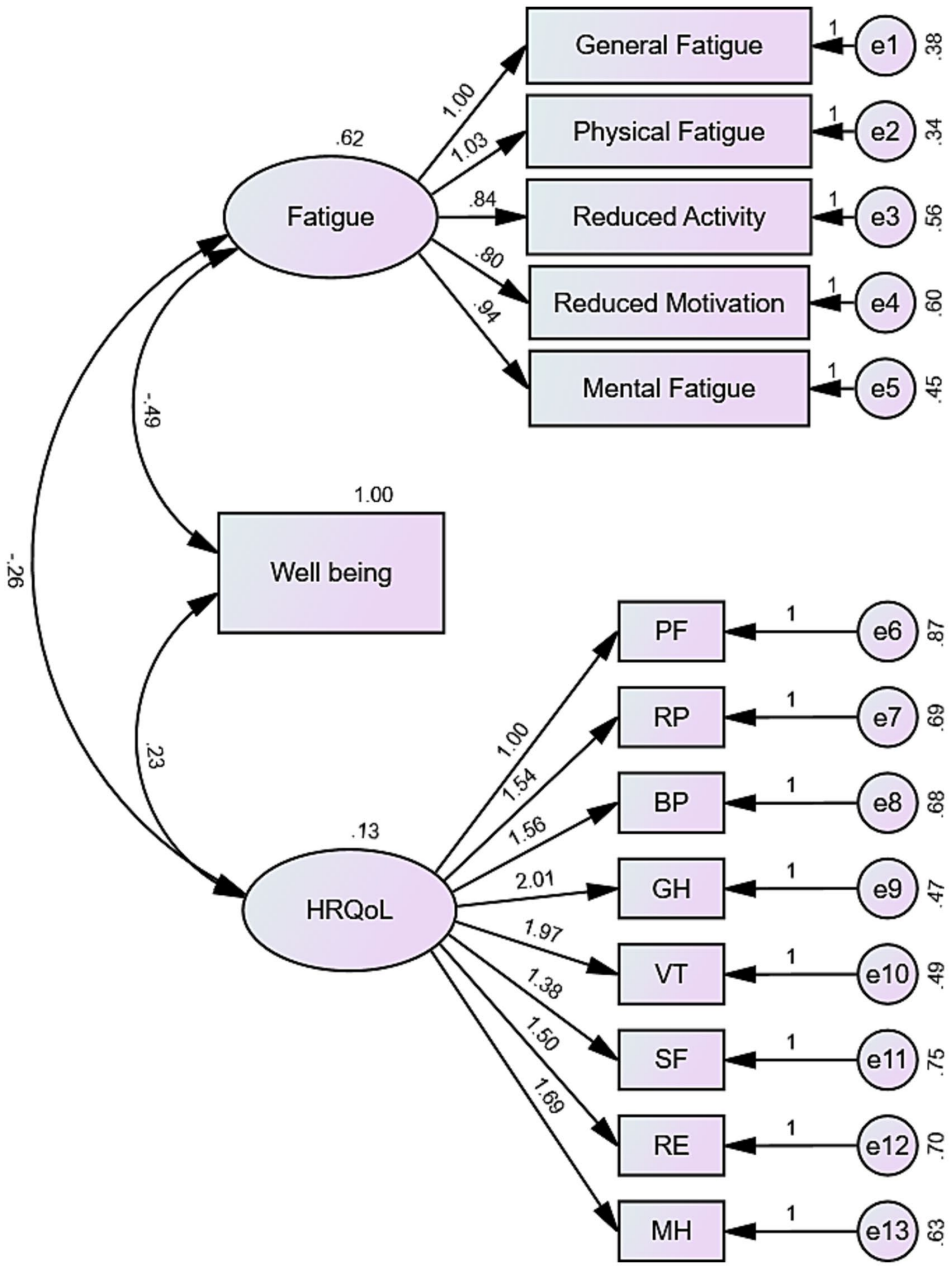
Structural equation model depicting the mediation mechanism of well-being. The model illustrates the relationships between fatigue, well-being, and HRQoL, with significance levels (*p* < 0.001). The model fit is reflected by the following indices: GFI = 0.880, NFI = 0.858, IFI = 0.884, TLI = 0.859, CFI = 0.884, RMSEA = 0.094.

To investigate these relationships further, we constructed a structural equation model (SEM) (Figure 4). The results highlighted that fatigue significantly affects well-being, with higher fatigue levels leading to lower well-being. In turn, lower well-being was associated with reduced HRQoL. Interestingly, fatigue also directly impacted HRQoL, with lower fatigue correlating with higher HRQoL. Importantly, well-being played a mediating role: while fatigue directly influenced HRQoL, it also indirectly affected HRQoL through well-being.

### 3.6 Independent risk factors associated with well-being score

Given the critical role of well-being in CHB patients, we analyzed independent factors related to well-being (Table 2). Our findings revealed that gender (OR = 2.692, *p* = 0.004), weight (OR = 0.971, *p* = 0.038), education level (OR = 0.642, *p* = 0.028), physical exercise (OR = 0.541, *p* < 0.001), and cirrhosis (OR = 1.944, *p* = 0.017) were independent factors influencing well-being in CHB patients. The logistic regression model demonstrated good fit, as confirmed by the Hosmer–Lemeshow test (*p* = 0.791).

**Table 2:** Multivariable analysis for well-being Index among CHB patients.

Variables	Bivariable analysis		Multivariable analysis	
	OR (95% CI)	*p* value	OR (95% CI)	*p* value
Age	1.012 (0.990–1.034)	0.287		
Gender	3.434 (1.957–6.023)	<0.001	2.692 (1.381–5.248)	0.004
Height	0.934 (0.902–0.968)	<0.001		
Weight	0.964 (0.942–0.987)	0.002	0.971 (0.945–0.998)	0.038
Marriage	1.309 (0.877–1.955)	0.188		
Education	0.521 (0.370–0.733)	<0.001	0.642 (0.432–0.953)	0.028
Physical exercise	0.534 (0.402–0.708)	<0.001	0.541 (0.398–0.735)	<0.001
Alcohol intake	1.251 (0.804–1.947)	0.322		
Cigarette smoking	1.015 (0.633–1.630)	0.950		
Cirrhosis	1.990 (1.244–3.184)	0.004	1.944 (1.126–3.356)	0.017
ALT levels	0.999 (0.989–1.010)	0.911		
LS levels	1.037 (0.993–1.083)	0.098		

ALT, Alanine Aminotransferase; LS, Liver Stiffness.

Additionally, we specifically examined the independent factors influencing well-being in CHB patients with cirrhosis (Table 3). The results identified only gender (OR = 5.884, *p* = 0.007) and physical exercise (OR = 0.541, *p* = 0.023) as the significant independent factors for this subgroup. The model for this subgroup showed acceptable calibration according to the Hosmer–Lemeshow test (*p* = 0.294).

**Table 3:** Multivariable analysis for well-being Index among CHB patients with cirrhosis.

Variables	Bivariable analysis		Multivariable analysis	
	OR (95% CI)	*p* value	OR (95% CI)	*p* value
Age	0.996 (0.952–1.041)	0.845		
Gender	5.333 (1.547–18.393)	0.008	5.884 (1.631–21.224)	0.007
Height	0.921 (0.866–0.979)	0.008		
Weight	0.944 (0.902–0.989)	0.015		
Marriage	0.511 (0.189–1.376)	0.184		
Education	0.547 (0.300–0.995)	0.048		
Physical exercise	0.571 (0.353–0.923)	0.022	0.541 (0.318–0.919)	0.023
Alcohol intake	0.844 (0.382–1.867)	0.676		
Cigarette smoking	1.032 (0.460–2.315)	0.939		
ALT levels	0.996 (0.973–1.020)	0.760		
LS levels	1.012 (0.954–1.074)	0.683		

ALT, Alanine Aminotransferase; LS, Liver Stiffness.

## 4 Discussion

This study included 428 patients with chronic hepatitis B (CHB), who were classified into high and low well-being groups based on WHO-5 scores. The aim was to identify independent factors associated with well-being. Our results showed that well-being scores were positively correlated with all eight dimensions of HRQoL, and negatively correlated with all five dimensions of fatigue. Further logistic regression analysis revealed that gender, body weight, educational level, physical exercise, and cirrhosis were independent factors associated with well-being among all CHB patients. However, in the subgroup of CHB patients with cirrhosis, only gender and physical exercise were identified as independent factors. Furthermore, we observed that while fatigue directly impacts HRQoL in CHB patients, well-being serves as a crucial mediating factor that indirectly links fatigue to HRQoL.

Well-being, often defined as long-term psychological functioning such as self-actualization and autonomy, has been extensively studied for its association with health outcomes (8). However, evidence remains inconsistent, with some studies reporting no significant associations between well-being and specific conditions, such as breast cancer or myocardial infarction (11, 13). At the same time, one study found no relationship between well-being and myocardial infarction, although higher well-being levels were associated with reduced risks of cancer, diabetes, and stroke (28). Notably, gender emerged as an independent factor associated with well-being in both the overall CHB population and the cirrhotic subgroup, with female patients reporting significantly lower well-being scores. This finding is consistent with prior research in chronic illness populations, where women are more likely to report psychological distress and lower levels of subjective well-being (29). Possible contributing factors include caregiving responsibilities, social expectations, and workplace challenges (30). In CHB patients, these may be further compounded by disease-related stigma. These findings highlight the need for gender-sensitive approaches in clinical care, particularly by strengthening psychological support for female patients.

Physical exercise was found to be independently associated with higher well-being scores in both the overall CHB population and the cirrhotic subgroup. This suggests that regular physical activity may serve as a protective factor for psychological health in CHB patients. Prior research has shown that exercise can reduce fatigue, improve sleep quality, and enhance self-efficacy—all of which may contribute to improved subjective well-being (31). In chronic disease populations, including patients with cancer, cardiovascular disease, and diabetes, exercise interventions have been associated with improvements in mood, emotional well-being, and quality of life (32, 33). Although limited, some studies have also reported similar benefits among patients with chronic liver diseases, including CHB. These findings highlight the potential value of integrating structured exercise programs into routine management of CHB patients (34). Future intervention studies are warranted to evaluate the effectiveness of exercise in improving well-being, reducing fatigue, and enhancing HRQoL in this population.

We also found that well-being plays an important mediating role in the relationship between fatigue and HRQoL. While fatigue directly affects HRQoL, well-being may mitigate this relationship by serving as an intermediary. This provides a theoretical foundation for improving HRQoL in CHB patients by addressing their levels of well-being scores. Despite advances in antiviral treatment with high genetic barriers to resistance, CHB patients still face prolonged, even lifelong, treatment duration (35, 36). The psychological burden of such therapies, coupled with the stigma associated with CHB, may exacerbate fatigue across various dimensions, disrupting patients’ daily lives and diminishing HRQoL. Since our research also revealed a complex reciprocal relationship between well-being and fatigue, where each factor significantly influences the other. It may indicate that improving well-being may alleviate fatigue and its impact on HRQoL. The underlying mechanisms remain to be clarified, but may involve improved emotional regulation, enhanced perceived social support, and reduced illness-related stigma. Future research should explore these potential pathways to better understand how well-being can be leveraged to improve health outcomes in this population.

This study had some limitations. Being a single-center study, the results may be biased. Additionally, the cross-sectional design precludes further causal inferences. Longitudinal or interventional studies are needed to confirm whether targeting well-being and reducing fatigue can directly improve HRQoL in CHB patients. Additionally, due to incomplete medication records, this study did not include data on medication types, adherence, or adverse events, which may influence well-being. This may have limited our ability to fully assess all relevant factors. Future studies should incorporate detailed medication data.

## 5 Conclusion

Our findings highlight the prevalence of low well-being among CHB patients and identify key risk factors, including female gender, low body weight, lack of physical exercise, lower educational levels, and cirrhosis. For CHB patients with cirrhosis, female gender and lack of exercise habits are the factors associated with low well-being. Fatigue is significantly negatively correlated with well-being, whereas HRQoL is positively correlated with well-being. While fatigue directly influences HRQoL, well-being serves as an essential mediator of its indirect effects. Interventions aimed at improving well-being, particularly through promoting physical activity, may hold promise for reducing fatigue and enhancing HRQoL in CHB patients.

## Data Availability

The raw data supporting the conclusions of this article will be made available by the authors, without undue reservation.
